# Update on the types and usage of liquid biopsies in the clinical setting: a systematic review

**DOI:** 10.1186/s12885-018-4433-3

**Published:** 2018-05-04

**Authors:** Borros Arneth

**Affiliations:** Institute of Laboratory Medicine and Pathobiochemistry, Molecular Diagnostics, University Hospital of the Universities of Giessen and Marburg UKGM, Justus Liebig University Giessen, Feulgenstr. 12, 35392 Giessen, Germany

**Keywords:** Liquid biopsy, Biomarker, Tumor marker, Cancer diagnostics

## Abstract

**Background:**

This systematic review aimed to gather evidence from research on the current state of liquid biopsy in medical practice, specifically focusing on mutation detection and monitoring.

**Methods:**

A systematic search was performed via Medline.

**Results:**

The results of this investigation indicate that liquid biopsy plays a critical role in the detection and management of tumors. This technique gives healthcare providers the ability to gather critical and reliable information that may potentially shape the diagnosis, treatment, and prognosis of a variety of cancers in the near future. This study further reveals that liquid biopsy has several potential shortcomings that may limit its application and use in the healthcare setting. Nevertheless, liquid biopsy remains a valuable tool that is gradually becoming a part of routine healthcare practice in oncology departments and hospitals worldwide.

**Conclusions:**

The evidence described herein reveals the potential relevance of liquid biopsy as an important prognostic, diagnostic, and theranostic tool. This non-invasive procedure enables healthcare practitioners to detect and monitor genomic alterations and will likely replace tumor tissue biopsy as the standard method for detecting and monitoring mutations in the future. The information obtained herein can enable physicians to make informed decisions regarding current treatment options; however, liquid biopsy has not yet been incorporated into routine clinical diagnostics for cancer patients.

## Background

The ability of healthcare practitioners to manage and treat diseases substantially depends on obtaining a precise diagnosis of the condition in question. As a result, the concept of precision medicine has evolved to become an important aspect of cancer treatment [1]. Currently, physicians and researchers work towards providing accurate cancer screening, diagnosis, and prognostication along with a correct prediction of recurrence and resistance to treatment [2–4]. The overall aim is to ensure that patients receive the best care for their condition, which will ideally return them to their former state of health [5].

Traditionally, physicians and researchers have regarded tissue biopsy as the gold standard for providing data that produces positive health outcomes among patients with a variety of cancers [6–9]. Furthermore, tissue biopsy provides the histopathological information that helps physicians determine whether a lesion is malignant [1, 10]. However, it is worth noting that commonly used tissue biopsy methods, such as surgical biopsy, are invasive, and their effectiveness depends on the cancer stage and local area that is biopsied [11]. In recent years, non-invasive techniques, such as liquid biopsy, have emerged as viable options to help physicians manage different types of cancerous cells [11, 12]. Research on the effectiveness of these non-invasive techniques, including that of liquid biopsy, must be performed to determine their usefulness for the detection and monitoring of genomic alterations. The objective of this systematic review was to collect evidence on the role and importance of liquid biopsy in mutation detection and monitoring.

## Methods

This study comprises an analysis of other studies that have examined the use of liquid biopsy in the detection and monitoring of mutations and cancer. Since this study used a secondary research approach, locating articles relevant to the research goal was imperative.

A manual search limited to English language articles published between 2008 and 2017 was performed in four electronic databases: PsycINFO, CINAHL, PubMed, and the Web of Science (CINAHL: Cumulative Index of Nursing and Allied Health). The search terms and phrases used to identify the articles relevant to the research topic included “liquid biopsy,” “status of liquid biopsy,” “role of liquid biopsy,” “liquid biopsy and mutation detection,” and “liquid biopsy and mutation monitoring.” In the CINAHL, for example, the terms “status of liquid biopsy,” “liquid biopsy,” “role of liquid biopsy,” and “liquid biopsy and mutation detection” were used to locate relevant studies, which yielded 37 relevant results.

The abstracts of the located articles were carefully assessed for their quality and appropriateness by examining the aim, research design, results, and conclusions of each selected article.

The data collection process involved summarizing the results and findings of the selected studies by focusing on the use of liquid biopsy for the detection and monitoring of tumors among cancer patients. Key data points included information on the type and category of liquid biopsy, the materials analyzed by the liquid biopsy procedure, and the liquid biopsy advantages and disadvantages. Since only evidence from previous studies was used to examine the role of liquid biopsy for tumor detection and monitoring, assessing the risk of bias in each study was imperative. This process included reviewing for systematic bias in the study design, findings, assumptions, and manner in which outcomes were used to determine conclusions.

The summary measures used in this review revolved around the role and effectiveness of various liquid biopsy techniques for accurately detecting and monitoring different types of tumor cells. The results of the selected studies were summarized, and comparisons were then performed to provide a clear portrait of the current state and importance of liquid biopsy.

## Results

**Fig. 1:**
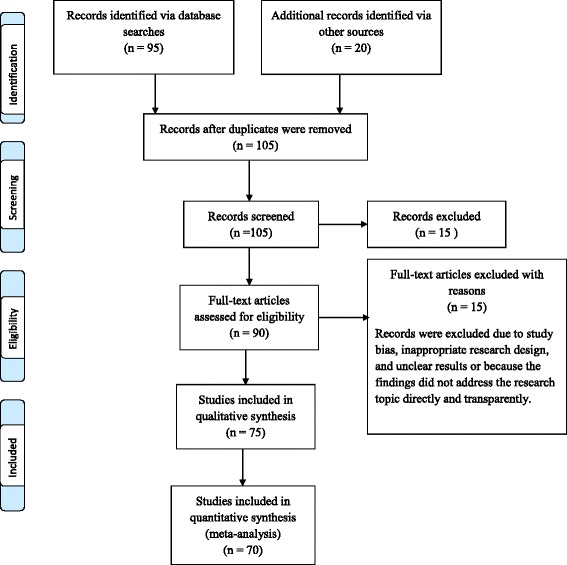
This figure shows the overall process from the literature search to data retrieval in the form of a PRISMA flow diagram

A total of 70 studies were used in this systematic review. Figure 1 depicts a flow diagram of the overall procedure from the literature search to data retrieval. Table 1 gives an overview about the studies that were selected at the end of the search process. The final set of articles consisted of longitudinal studies, randomized control studies, meta-analyses, and systematic reviews. Using a variety of study designs implied that each study possessed a unique risk for bias. Nevertheless, the articles that were used provided valuable and reliable information that helped in determining the current status of liquid biopsy. More specifically, the selected studies focused on liquid biopsy procedures, the types and categories of liquid biopsy, the materials analyzed in liquid biopsy procedures, and liquid biopsy advantages and disadvantages. A synthesis of the articles revealed that previous studies have considered liquid biopsy to be a valuable method for gathering information for tumor detection and monitoring.

**Table 1:** Studies that were selected at the end of the search process

Study	Purpose	Design	Summary of findings
Heitzer, Ulz and Geigl (2015) [38]	To examine the use of cell-free DNA in liquid biopsy	Systematic review	The study reported that cell-free DNA is usually released from tumor locations and inflamed tissues as cells undergo necrosis or apoptosis. This form of tumor-derived DNA can be extracted from plasma and used to detect B-Raf proto-oncogene, KRAS, and serine/threonine kinase V600E mutations.
Molina-Vila et all. (2016) [14]	To examine the state of different cell operation and isolation techniques that have been used in liquid biopsy	Systematic review	The study reported that CTCs have played a key role in the management of cancer. In addition, the study showed that CTCs are used as critical pharmacodynamic and prognostic biomarkers for the management and diagnosis of cancer.
Torre et al. (2015) [16]	To review global cancer statistics	Systematic review	The researchers reported that the incidence and impact of cancer are more pronounced in developing nations than in developed countries. In addition, developing countries account for 57 and 65% of cancer cases and deaths worldwide.
Cronin et al. (2009) [18]	To examine the rates of breast cancer in the US	Systematic review	Cancer is a significant and common health problem affecting millions of Americans.
Mayo-de-Las-Casas et al. (2018) [19]	To examine methods for analyzing circulating free DNA	Systematic review	There are a wide range of methods and techniques for analyzing circulating free DNA.
Bettegowda et al. (2014) [20]	To explore the detection of circulating tumor DNA among patients with early and late malignancies	Systematic review	Liquid biopsy helps in detecting malignancies in the human body.
Braig et al. (2015) [21]	To explore the emergence and detection of epitope-changing mutations	Cohort study	Liquid biopsy can be used to detect EGFR G465R mutations.
Carvalho and Oliveira (2014) [22]	To identify biomarkers of cancer	Systematic review	Extracellular vesicles are critical markers of cancer.
Dawson, Rosenfeld, and Caldas (2013) [23]	To examine the role of circulating tumor DNA in cancer detection	Systematic review	Circulating tumor DNA is an important and reliable biomarker of metastatic breast cancer.
de Bruin et al. (2014) [24]	To examine the spatial and temporal genomic changes associated with non-small cell lung cancer	Systematic review	Non-small cell lung cancer was linked to intratumor heterogeneity in translocations, copy number alterations, and APOBEC cytidine deaminase activity.
Diaz et al. (2012) [25]	*To examine whether KRAS* DNA could be identified and detected in patients receiving a therapeutic anti-EGFR antibody	Longitudinal study	*KRAS* DNA was detected in 38% of the patients.
Crowley et al. (2013) [26]	To study how liquid biopsy can be used to detect and diagnose cancer	Systematic review	Liquid biopsy serves an effective tool for diagnosing cancer and analyzing the response to treatment.
Diaz et al. (2016) [27]	To explore the use of liquid biopsy in sensitive mutation detection	Longitudinal study	BEAMing and Safe-SeqS are effective in the detection of sensitive mutations among cancer patients.
Tu et al. (2016) [29]	To study the analytical strategies utilized in the assessment of oncogenic mutations	Systematic review	An EFIRM-based liquid biopsy can accurately detect oncogenic mutations.
El Messaoudi et al. (2013) [30]	To study the preanalytical parameters that affect ccfDNA concentration	Systematic review	The researchers determined an optimal preanalytical protocol that can be used in liquid biopsy procedures.
Janku et al. [2015] [31]	To compare mutations detected in plasma cfDNA and archival tissue	Cohort study	The outcomes were similar for the plasma cfDNA and archival tissue in 91% of the cases.
Liu et al. (2013) [32]	To study the diagnostic accuracy of pleural effusion and tumor tissue	Randomized control trial	MPE and plasma are effective materials for EGFR mutation detection in patients with lung cancer.
Brevet et al. (2011) [33]	To explore the use of spectrometry genotyping in EGFR mutations detection	Longitudinal study	Mass spectrometry genotyping is a feasible method for detecting EGFR mutations.
Zhao et al. (2013) [34]	To examine growth factor receptor mutation statuses in patients with lung cancer	Longitudinal study	Growth factor receptor mutations are critical indicators of lung cancer.
Lim et al. (2017) [35]	To explore the biomarkers of metastatic colorectal cancer	Systematic review	NRAS, KRAS, BRAF, PTEN, and PIK3CA were effective biomarkers of metastatic colorectal cancer.
Guttery et al. (2013) [40]	To identify the indicators of breast cancer	Systematic review	cfDNA and microRNA can be used to identify breast cancer.
O’Flaherty et al. (2012) [41]	To examine the clinical utility of circulating tumor cells	Systematic review	Circulating tumor cells can help in the diagnosis of lung cancer.
Mead et al. (2012) [42]	To study the biomarkers of normal colons, cancers, and benign polyps	Systematic review	Circulating tumor markers can help in the identification of normal colons, cancers, and benign polyps.
Zandberga et al. (2013) [43]	To study the prognostic, predictive and diagnostic values of cell-free microRNAs	Systematic review	Cell-free microRNAs can be used in predicting and diagnosing lung cancer.
Martinez-Galan et al. (2014) [45]	To analyze ER expression statuses in cancer patients	Longitudinal study	*ESR1* influences estrogen receptor protein expression in patients with cancer.
Krebs et al. (2010) [36]	To explore the utility of circulating tumor cells in the management of cancer and predicting outcomes	Systematic review	Molecular characterization of the tumor cells provides a unique ability to assess and monitor the phenotypic and genotypic features of any cancer without the need to carry out an invasive biopsy. Thus, systems such as CellSearch, flow cytometry, immunofluorescence-based technologies, and reverse transcriptase PCR can help analyze tumor cells.
Regalado (2014) [37]	To examine how cancer can be spotted in a vial of blood taken from patients	Randomized controlled trial	Using a sample population of 846 patients suffering from 15 different cancer types, the researcher showed that analyzing tumor cells helped detect the presence of metastatic tumors in 80% of patients. Additionally, the researchers reported that the tumor cell analysis did not produce any false positives in the sample population.
Cree (2015) [39]	To examine the clinical utility as well as the cost-effectiveness of liquid biopsy in cancer treatment and management	Systematic review	Circulating tumor cells, circulating tumor DNA, and other protein markers enable caregivers to use liquid biopsy for the detection and monitoring of actionable RASSF1A mutations in patients with lung cancer.
Han, Wang, and Sun (2017) [44]	To examine the role of circulating tumor DNA as a biomarker in cancer detection	Systematic review	Circulating tumor DNA can be used to measure the tumor load and detect EGFR, PIK3CA, BRAF, KRAS, HER2, ALK, PDGFR, and KIT mutations in multiple cancer types, such as cancer of the lungs, pancreas, breast, prostate, and colorectal region. In addition, a considerable amount of research evidence supports that ctDNA can help in identifying mutations that are related to treatment choices with high specificity and sensitivity.
Piva (2015) [47]	To study the use of exomes in liquid biopsies	Systematic review	The researchers reported that exosomes have double-stranded DNA of the parent cells that can be analyzed in liquid biopsies.
Mayo-de-Las-Casas et al. (2017) [51]	To examine the role of liquid biopsy in the diagnosis and management of non-small cell lung cancer	Systematic review	miRNAs are non-coding types of RNA molecules that regulate the gene expression process. A single miRNA can influence the expression of several genes in the human body. Additionally, miRNAs are typically distributed throughout the entire human genome. However, most miRNAs are found in fragile sites that are quickly deleted by the existence of different types of cancers.
Spigal et al. (2016) [56]	To explore the total mutation burden in lung cancer and examine its relationship with the response to various PD-1/PD-L1-targeted therapies	Clinical trial	The assessment and monitoring of tumor mutations are very critical in the management of lung cancer. Mutations often lead to poor prognosis and high disease burden on patients.
Guibert et al. (2015) [58]	To explore KRAS mutations and their prognostic impact during treatment	Meta-analysis	The study revealed that higher levels of KRAS cfDNA allele mutations were correlated with poor responses to treatment. Thus, the collection of evidence related to cell mutations is critical in the treatment and monitoring of cancer [56, 57, 59].
Mardis and Wilson (2009) [60]	To explore the clinical significance and effectiveness of cancer genome sequencing	Systematic review	DNA sequencing with the Sanger method can identify structural variant loci, copy number, and focal mutations (such as small in/dels and single nucleotides) in the tumor genome.
Malapelle et al. (2016) [63]	To examine the use of next-generation sequencing techniques in liquid biopsies involving non-small cell lung cancer patients	Systematic review	The next-generation sequencing method, on the other hand, relies on the capillary electrophoresis technique to fragment genomic strands and identify bases in each fragment via emitted signals. The technique helps in the detection of ARID1A, SOX9, FAM123B, ATM KRAS, PIK3C, and TP53 mutations.
Østrup et al. (2017) [64]	To examine the use of a liquid biopsy to determine somatic copy number alterations (SCNAs)	Clinical trial	The study showed that the somatic number of alterations that occur in tumors provide critical information on tumor classification, treatment targets, and patient responses to cancer treatment interventions, such as chemotherapy. Thus, copy number alterations can be assessed using plasma samples that contain cell-free tumor DNA (ccfDNA).
Wang et al. (2017) [65]	To explore the use of liquid biopsy in the detection of early- and late-stage human malignancies	Cohort study	A liquid biopsy is a less invasive test that can frequently be performed with less morbidity than conventional biopsies [65]. This feature is of great importance when healthcare practitioners want to provide temporal measurements of the tumor burden and analyze the possibility of recurrence and malignancy.

### Liquid biopsy in Cancer research and literature

The review of the retrieved articles revealed that liquid biopsy has been an important topic for cancer research [13–15]. Torre et al. noted that cancer is a leading cause of mortality worldwide [16]. The burden of the condition is most pronounced in aging populations in both developed and developing nations. Some of the most commonly diagnosed types of cancer include prostate, colorectal, cervical, bladder, lung, liver, and stomach cancer.

Torre et al. showed that the incidence and impact of cancer are more pronounced in developing nations than in developed countries, with developing countries accounting for 57% of cancer cases and 65% of cancer deaths worldwide [16]. This phenomenon is attributable to the effects of competing causes of mortality, such as infections [16]. The burden of cancer affects patients, their families, governments, and society as a whole. In addition, cancer significantly impacts the healthcare sector [17, 18]. Consequently, researchers have been striving to develop more effective methods for managing and treating cancer, and liquid biopsy is widely considered a viable and promising means to facilitate cancer management worldwide [13–15].

Molina-Vila et al. described liquid biopsy as a minimally invasive test that healthcare practitioners can use to assess the genetic status of cancer tumors by analyzing cell materials, such as free DNA, exosomal DNA, microRNA (miRNA), and tumor cell DNA [16]. Since liquid biopsy procedures involve the use of blood samples, which are easy to obtain, this technique is widely regarded as useful when compared to traditional biopsy [19–21].

The goal of a liquid biopsy test is to identify the materials in a sample that originate from cancerous cells [16]. Cancerous cells release biomarkers and other information in the form of cell fragments and dead cells or via tumor cell necrosis. In most cases, the phagocytes that process cellular DNA will engulf cell fragments and the dead cells that originate from a tumor [20, 22].

Since these materials come directly from cancerous cells, they can be tested and examined for genetic aberrations similar to those found in tumors. These genetic aberrations may appear via chromosomal rearrangement, amplification, mutation, and hypermethylation [23–25]. The liquid biopsy process involves blood collection from the patient, cell removal, and plasma isolation [26–28]. The plasma is then superheated, and the resulting material is analyzed [29, 30].

Molina-Vila et al. remarked that liquid biopsy has been incorporated into conventional clinical practice in oncology departments and hospitals that treat lung, melanoma, breast, ovarian, cervical, and bladder cancer [14]. The authors added that the adoption of this technology in hospitals has already improved the manner in which healthcare practitioners detect and monitor KRAS, BRAF, and EGFR mutations in patients with lung, colon, and breast cancer, respectively [14]. While the test has yet to reach its full potential, liquid biopsy is quickly becoming a standard oncological tool for obtaining valuable prognostic, predictive, and diagnostic information on KRAS, BRAF, and EGFR mutations [14, 27].

In most cases, the liquid biopsy procedure is performed by a physician who carefully collects a sample that can be used to obtain the relevant information regarding cancerous cells. Molina-Vila et al. explained that samples collected during a liquid biopsy contain different cell fragments, such as exosomes, circulating tumor cells, tumor-educated platelets, and circulating tumor DNA [14]. These different materials and DNA sources provide unique and significant opportunities for physicians to analyze KRAS, BRAF, and EGFR mutations. In addition, they help physicians to understand the characteristics and nature of other tumors, such as adenomas, fibromas, and meningiomas [14, 31–33]. Overall, the liquid biopsy process improves the diagnostic, prognostic, and preventive aspects of providing healthcare to patients [14, 34].

## Discussion

The evidence from the research collected from these selected studies reveals the potential of liquid biopsy as an important prognostic, diagnostic, and theranostic tool. In particular, liquid biopsy is a non-invasive tool that accurately detects and monitors genomic alterations. The procedure can be used to identify a broad range of mutations, including KRAS, BRAF, and EGFR mutations, in patients with colon carcinoma, breast cancer, melanoma, and lung cancer. Although the current study provides essential research evidence that may be used to help improve cancer management, it is imperative to note that this study lacks its own statistical strength because the findings and conclusions are entirely based upon the outcomes of previous studies. However, based upon these prior studies, the current study demonstrates that liquid biopsy can play a critical role in cancer management via its ability to accurately detect and monitor tumors. Therefore, liquid biopsy will likely replace tumor tissue biopsy in the future as the standard tool for detecting and monitoring mutations.

### Types and categories of liquid biopsies

There are two different test categories for liquid biopsies. The first category is based on the type of material analyzed in the test, while the second is based on the type of analysis that is performed to detect or monitor cancer.

### Categorization based on material type

#### Analysis of tumor cells

One material that can be analyzed to obtain critical information on genetic aberrations is tumor cells. This test identifies the presence of tumor cells in the bloodstream and can be used for the management of all cancer types. According to Krebs et al., the molecular characterization of tumor cells provides a unique method for assessing and monitoring the phenotypic and genotypic features of any cancer without requiring an invasive biopsy [35].

Researchers have described several systems that facilitate tumor cell analysis, such as CellSearch, flow cytometry, immunofluorescence-based technologies, and reverse transcriptase PCR. For example, the FDA has approved the CellSearch system (Menarini-Silicon Biosystems Inc., Menarini Group, Florence, Italy) for clinical use in testing for circulating tumor cells in cancer patients [35, 36]. This particular approach is based on image cytometry and semi-automated immunomagnetic enrichment. Tumor cells are detected and then analyzed using an antibody that bonds to epithelial cell adhesion molecules (EpCAM) [31]. This method along with other isolation methodologies can help determine the aggressiveness of cancers.

In a recent study by Regalado that used a sample of 846 patients with 15 different types of cancer, tumor cell analysis helped to detect the presence of metastatic tumors in 80% of the patients [37]. Furthermore, the researchers reported that the tumor cell analysis did not produce any false positives in the sample population [37].

### Analysis of free DNA

Free DNA is another material that can be analyzed in liquid biopsies [23]. According to Heitzer et al., cell-free DNA (cfDNA) is usually released from tumors and inflamed tissues as cells undergo necrosis or apoptosis [38]. This tumor-derived DNA can be extracted from plasma and used to detect BRAF proto-oncogene, KRAS, and serine/threonine kinase V600E mutations. In a study by Cree, circulating tumor cells, tumor DNA, and other protein markers reportedly enabled physicians to use liquid biopsy for the detection and monitoring of actionable RASSF1A mutations in patients with lung cancer [39].

Heitzer et al. further noted that both manual and automated systems exist for extracting DNA from plasma, such as those marketed by Siemens, Qiagen, and Promega [39]. Some researchers have reported that the ability to detect circulating tumor cells, particularly in low-volume samples, remains a concern for the use of liquid biopsy among cancer patients [40–42]. This explains why circulating free tumor DNA (ctDNA, cfDNA) continues to be an essential blood-based biomarker typically analyzed by liquid biopsy [39, 43].

Han et al. stated that circulating tumor DNA could be used to measure the tumor load and detect EGFR, PIK3CA, BRAF, KRAS, HER2, ALK, PDGFR, and KIT mutations in multiple cancer types, such as lung, pancreas, breast, prostate, and colorectal cancer [44]. Additionally, a considerable amount of evidence from research indicates that circulating tumor DNA can be used to precisely identify mutations that require specific treatment options with high specificity and sensitivity [39, 45, 46]. Although alterations in copy number may be difficult to analyze, the process has been successful in some cases but at considerable cost [44].

### Analysis of Exosomal DNA

Exosomal DNA can also be analyzed via liquid biopsies. Recent studies have shown that exosomes contain the double-stranded DNA of their parent cells [47]. This discovery coupled with the possibility of isolating exosomes originating from specific tissues and tumors indicates that exosomal DNA can be used to assess tumor heterogeneity in various carcinomas, such as those in the lung, breast, and prostate cancer.

Gold et al. asserted that the use of highly sensitive technologies, such as digital PCR, can improve the reliability of exosomal DNA analysis by amplifying exosomes during testing [48]. Furthermore, these techniques demonstrate that exosomal DNA analysis can be used to evaluate the selective effects of therapies, such as chemotherapy, on cancer cell populations [48–50].

### Analysis of miRNAs

Another type of liquid biopsy analysis involves miRNAs. According to Molina-Vila et al., miRNAs are non-coding RNA molecules that regulate gene expression [51]. A single miRNA can influence the expression of several genes in the human body. Additionally, while miRNAs are typically distributed throughout the human genome, most miRNAs are found in fragile sites that are quickly deleted by different types of cancer [51, 52]. Therefore, miRNA alterations are able to reflect cancer progression and development. The most common markers that show miRNA alterations in patients with colorectal, lung, breast, and skin cancer include miR-21, miR-320a, miR-423-5p, and miR-24 [51–53]. Other markers, such as miR-106a, miR-29a, and miR-106b, have also been shown to be effective for the diagnosis of colorectal cancer.

Although the role of miRNAs in the cause and progression of cancer is known, no techniques for the analysis of miRNAs as a liquid biopsy post-processing procedure have provided promising results. The systematic literature search conducted by this study did not identify any research that demonstrated practical, promising results for using miRNAs as a liquid biopsy diagnostic tool.

### Categorization based on analysis type

#### Small-scale mutation analysis and targeted deep sequencing

A primary analysis that can be performed via liquid biopsy is detecting small-scale mutations (point mutations, small defined insertions, and small defined deletions). From a clinical perspective, the detection of EGFR, BRAF, KRAS, and KIT mutations is important because it can provide vital information on when to start certain treatments. For example, EGFR antibody therapy can be initiated only if there are no mutations in the RAS gene [6, 7]. Currently, the standard procedure for determining the presence of RAS gene mutations is tumor tissue biopsy. Since previous studies have demonstrated the effectiveness of liquid biopsy in detecting EGFR, PIK3CA, BRAF, KRAS, HER2, ALK, PDGFR, and KIT mutations, liquid biopsy procedures may replace tumor tissue biopsy for detecting these mutations in the future [6, 8, 44, 54, 55].

Another advantage of the use of liquid biopsy for mutation detection and monitoring is that it can provide essential information on the prognosis, resistance, and disease burden [56, 57]. According to Spigal et al., assessing and monitoring tumor mutations are critical for managing lung cancer. Certain mutations often lead to poor prognoses and high disease burdens for patients [56]. Guibert et al. studied KRAS mutations and their corresponding prognostic impacts during treatment [58]. The study revealed that higher levels of KRAS cfDNA allele mutations correlated with poorer responses to treatment. Therefore, the collection of information related to cell mutations is critical for treating and monitoring cancer [56, 57, 59]. Furthermore, modern techniques have enabled the high-sensitivity deep sequencing of important small mutations (insertions, deletions, and point-mutations).

### Analysis of structural changes

Analyzing structural changes using the Sanger method is another common type of analysis performed via liquid biopsy. This type of DNA analysis has been commercialized by Applied Biosystems and is based on the careful and selective incorporation of chain-terminating dideoxynucleotides during in vitro DNA replication. Studies have shown that DNA sequencing using the Sanger method can identify structural variation loci, copy number variations, and focal mutations, such as small insertions/deletions (ins/dels) and single nucleotide polymorphisms (SNPs), in a tumor genome [60, 61]. Furthermore, this method has helped advance the understanding and analysis of genes carrying somatic mutations of different cancer types, such as glioblastoma multiforme and lung adenocarcinoma [60].

### Large-scale mutation analysis by next-generation sequencing

The next-generation sequencing method uses the capillary electrophoresis technique to fragment genomic strands and then identify bases in each fragment via their emitted signals [62]. This technique facilitates the detection of ARID1A, SOX9, FAM123B, ATM KRAS, PIK3C, and TP53 mutations [62]. Next-generation sequencing is evolving into a reliable standard technique that can also be used to analyze cfDNA to carefully select lung, skin, colon, and breast cancer patients for tyrosine kinase inhibitor treatment. Furthermore, this method can also monitor the effects of tyrosine kinase inhibitors and other treatments by detecting the T790 M point mutation and EGFR exon 19 deletions [63]. The notable advantage of this methodology is that it enables the sequencing of entire genes and/or complete cfDNA.

### Analysis of copy number alterations

Finally, liquid biopsy can be used to analyze alterations in copy number. According to Østrup et al., the number of somatic alterations that occur in tumors provides critical information regarding tumor classification, treatment targets, and a patient’s potential response to cancer treatments, such as chemotherapy [64]. Copy number alterations can be assessed using plasma samples that contain cfDNA. However, it is worth noting that the detection of copy number alterations and biallelic losses via cfDNA is dependent on the tumor-derived content of the plasma sample [64]. Nevertheless, cfDNA can provide information on copy number alterations by reflecting changes in CCNB2 and CDKN2A/B in patients with lung, brain, breast, and skin cancer [64].

### Advantages and disadvantages of liquid biopsy

#### Advantages of the liquid biopsy procedure

As with any cancer intervention, liquid biopsy has several strengths that support its use in the treatment and management of a variety of cancer types. The first strength is that liquid biopsy is a minimally invasive technique. According to Bettegowda et al., liquid biopsy is a less invasive technique that can be performed more frequently and with lower morbidity than conventional biopsy [65]. These features are especially important for taking temporal measurements of tumor burden and for analyzing the possibility of recurrence and malignancy [65]. Additionally, available research evidence has shown that liquid biopsy can provide a better reflection of all the tumor profiles and genetic mutations in a patient [66].

Ilié and Hofman noted that liquid biopsy provides accurate genetic information for the investigation and analysis of essential companion diagnostics and genetic mutations [66]. They also note that liquid biopsies have clear advantages over conventional tissue biopsies because they are a source of fresh and reliable tumor-derived cell components and materials, and the materials are not contaminated by any form of preservative [66]. Recent studies that have compared the effectiveness and clinical value of liquid biopsy versus tissue biopsy have reported that the former is more clinically valuable based on its ability to accurately detect ARID1A, SOX9, FAM123B, ATM KRAS, PIK3C, and TP53 in patients suffering from a variety of cancers [64–66].

Another strength of liquid biopsy is its sensitivity and high predictive power. Mok et al. conducted an exploratory analysis of the clinical significance of blood samples and matched these samples with tumor samples from mutation testing performed among non-small cell lung cancer (NSCLC) patients [67]. The researchers randomized patients to receive gemcitabine/platinum and sequential erlotinib or a placebo in six cycles. Mutation testing was performed using Cobas Blood and Tissue Tests [67].

The results showed that the concordance between the blood and tissue tests was 88%. Moreover, the study reported that the blood-based test was highly specific and more sensitive than the tissue-based test [67].

Finally, liquid biopsy has been demonstrated to provide information regarding tumor burden in patients with lung, breast, colon, skin, and prostate cancer. Newman et al. examined the role of liquid biopsy in mutation detection and disease burden monitoring by quantifying cfDNA levels and comparing these data with those from positron-emission tomography (PET) and computed tomography (CT) tumor images [68]. The study focused on patients with NSCLC. The study demonstrated a positive correlation between cfDNA levels and tumor volumes as measured by PET and CT (R_2_ = 0.89, *P* = 0.0002) [68]. Additionally, the study reported that lower plasma cfDNA levels correlated with better clinical outcomes for patients [68].

Although some studies have not been able to demonstrate a positive correlation between the cfDNA level and tumor volume as measured by PET, some available research provides evidence that liquid biopsy can help determine tumor burden and has significant prognostic value because of its ability to detect KRAS, FAM123B, ARID1A, BRAF, and EGFR mutations [64–66].

### Disadvantages of the liquid biopsy procedure

A critical review of previous studies has revealed that liquid biopsy, similar to any other test, has limitations that affect its application. Furthermore, these limitations may impact the ability of physicians to rely on blood-based test results and thus effectively identify and manage mutations. One of the main shortcomings of liquid biopsy is that variations in cfDNA levels among patients may compromise the accuracy and reliability of the tests [14].

Previous studies have shown that the levels of cfDNA in serum and plasma vary [14, 69, 70]. Among cancer patients, tumor-derived cfDNA accounts for 0.1 to 10% of the total cfDNA [14]. Additionally, the level of tumor cfDNA depends on a variety of factors, such as the cancer stage, tumor vascularization, tumor burden, metastatic potential of cancerous cells, and apoptosis rate [28, 69].

Variations that occur in cfDNA levels with disease burden and stage suggest that some patients suffering from the early-stage disease will not have sufficient cfDNA to facilitate accurate testing. In some cases, tumor-derived cfDNA accounts for less than 1% of the total circulating DNA [14]. Therefore, physicians may encounter difficulty in gathering the diagnostic, prognostic and preventive information needed for the effective management of cancer.

The fact that typical genomic DNA can also be found in cfDNA is another matter of concern for researchers and healthcare practitioners who would use liquid biopsy in the management of cancer. Moreover, liquid biopsies may only identify specific mutations within a limited number of loci within a single gene [14, 28]. In these cases, physicians require highly sensitive approaches and techniques to sequence and identify mutations [68–70]. In addition, ultrasensitive detection and identification of mutations require extensive validation to ensure that the results are clinically actionable [14, 70]. Despite these shortcomings, the research analyzed during the current study demonstrates that liquid biopsy can play a key role in the identification, diagnosis, treatment, and management of cancer.

### Infrastructural challenges limiting liquid biopsy as an alternative to tumor biopsy

Currently, liquid biopsy is too expensive to use as a routine laboratory technique. The associated costs include those for equipment, extremely expensive reagents for high-throughput sequencing, labor, and professional costs for biochemists, bioinformatics specialists, and physicians. Therefore, the current costs of liquid biopsies are substantially higher than those for comparable conventional biopsies.

Reliability is another matter of concern. Cancer is a dire diagnosis. Therefore, patients and physicians expect and require accuracy from diagnostic tests. Liquid biopsy has not yet reached the level of validity required for widespread implementation in routine clinical diagnostics.

Additionally, biochemists, bioinformatics specialists, and physicians will need to work together as a co-operative team to advance the application of liquid biopsy. Initially, conventional biopsy and liquid biopsy will need to be performed in parallel to investigate and evaluate the potential of liquid biopsy for tumor diagnostics, which would require numerous studies of individual patients. Unfortunately, conducting a large number of studies will result in much greater cost for equipment, labor, reagents, and professional expenses.

In particular, the post-processing lab work needed for bioinformatics and statistical analyses is much simpler, cheaper, and well established for conventional pathology than for liquid biopsy post-processing. Liquid biopsies require more complex bioinformatics to process, compare, and interpret the identified sequences and mutations.

Consequently, although a role for liquid biopsy has already been demonstrated for some cancers in studies using small samples, its application and effectiveness as a therapeutic tool for all cancers require further validation.

Currently, cancer is diagnosed by conventional biopsies and subsequent histopathology and microscopy, and liquid biopsy is used only for research purposes or to supplement conventional pathology, e.g., to evaluate tumor sensitivity to certain drugs and/or for treatment monitoring.

### Differences between the conventional sequencing of tumor tissue and liquid biopsy

Tumor tissue is very heterogeneous genetically, and tumor cells are therefore not all genetically identical to one another. The DNA sequencing of tumor tissue provides only the sequence of the predominant tumor cell. Modern high-throughput techniques (such as Illumina) enable the identification of several predominant tumor cells but not of all tumor cells unless every tumor cell has been sequenced. Consequently, some important tumor cells may not have been identified by the sequencing of tumor tissue. However, these cells may be identified by liquid biopsy. It is worth noting that tissue and liquid biopsies investigate different parameters and therefore do not deliver identical results.

### Using cfDNA to monitor Cancer progression

Due to the characteristics of liquid biopsy, cfDNA will likely be used in the near future to monitor tumor alterations and tumor progression, much like classical tumor markers are used now.

If a cancer patient develops quantitative and/or qualitative alterations in serum cfDNA, it is likely that the tumor has changed. The emergence of other/new mutations in the cfDNA of a cancer patient is a clear indication that the tumor is changing and developing new mutations as well. Similar changes occur if the quantity of cfDNA changes. Decreased serum cfDNA after chemotherapy indicates that the treatment worked. By contrast, increased serum cfDNA indicates that the tumor is growing. More studies are required to confirm these assumptions.

## Conclusion

The evidence from the research collected in this systematic review reveals the potential relevance of liquid biopsy as an important prognostic, diagnostic, and theranostic tool. This non-invasive method enables healthcare practitioners to detect and monitor genomic alterations, and the available research evidence indicates that the test has been used successfully among patients with colon carcinoma, breast cancer, melanoma, and lung cancer. Nevertheless, healthcare practitioners and organizations have been slow to embrace the technique due to several important factors, such as the lack of a multiplicity of relevant biomarkers, turnaround time, and variations in cfDNA levels at different stages of disease. However, researchers agree that liquid biopsy represents a potentially major new strategy that healthcare providers can use to detect, treat, and monitor mutations and cancer, but further studies are required to address the limitations of this technique.
